# Pediatric Tuina in children with autism spectrum disorder: a study protocol for a randomized controlled trial

**DOI:** 10.1186/s13063-022-06030-4

**Published:** 2022-01-25

**Authors:** Xiang Feng, Quanrui Jiang, Yuxing Zhang, Tao Li, Wei Wei, Jun Yu, Wu Li, Jiangshan Li

**Affiliations:** School of Acupuncture-Moxibustion and Tuina, Hunan University of Chinese Medicine, Changsha, 410208 Hunan China

**Keywords:** Autism spectrum disorder, Pediatric Tuina, Randomized controlled trial

## Abstract

**Background:**

Autism spectrum disorder (ASD) is a neurodevelopmental condition characterized by repetitive stereotypical behavior and communication deficits. Currently, it lacks a specific clinical treatment method. Pediatric Tuina is a recent therapy in traditional Chinese medicine (TCM) and has been used to treat children with ASD. Nonetheless, it remains uncommon given the lack of large-scale evidence-based medical studies. This study aims to compare the efficacy of Tuina and conventional treatment in children with ASD.

**Methods:**

Eligible children will be randomly assigned to either the pediatric Tuina plus conventional treatment group or to the conventional treatment alone group based on a random table at a ratio of 1:1. The effectiveness of the Tuina intervention for ASD will be evaluated by a third-party organization. The pre- and post-intervention scores on the Childhood Autism Rating Scale comprised the primary outcome, whereas pre- and post-intervention scores on the Autism Treatment Evaluation Checklist were the secondary outcomes to assess improvement in symptoms. Baseline values of the participants will be determined at the time of registration. Outcomes will be evaluated after the 30th treatment session. The follow-up period will last for 6 months after treatment.

**Discussion:**

This study will evaluate the effectiveness and safety of Tuina in the treatment of ASD. The results of this study could provide reliable evidence to improve the management of patients with ASD.

**Trial registration:**

Chinese Clinical Trial Registry (*CHICTR*), ChiCTR2000040452. Registered on 28 November 2020.

## Background

Autism spectrum disorder (ASD) is a neurodevelopmental disorder characterized by typical repetitive behaviors and communication difficulties [1]. It has a childhood onset, and patients with this condition have lifelong difficulties with social interaction, communication, and sensory perception [2]. The etiology of ASD involves complex and polygenic interactions as well as possible environmental factors [3]. ASD is a serious public health problem worldwide, with its high morbidity and disability rates garnering increasing attention. Based on the epidemiological studies, the incidence rate of ASD is > 1–2% [4].

However, there are no specific agents for the treatment of ASD, which is currently treated with behavioral interventions such as early parent-mediated interventions [5–8]; naturalistic behavioral developmental interventions; behavioral and social treatments for school-age children, adolescents, and adults [9–12]; and even medical clown interventions [13]. These clinical interventions can significantly improve the social adaptability of children with ASD.

Clinical treatment of ASD using evidence-based pharmacology is limited to treating the co-occurring behavioral problems caused by mental illness rather than ASD itself. Risperidone [14–17] and aripiprazole [14, 18, 19] have been approved by the Food and Drug Administration to improve irritability or restlessness in children and adolescents with ASD. However, both drugs can cause adverse effects, including sedation and weight gain after long-term use, which increases the risk of subsequent health problems [19].

To alleviate the clinical symptoms of ASD and to save medical costs, traditional therapies have been used in the treatment of ASD with numerous advantages.

Tuina is an important and effective external treatment in improving stunting [20] and decreasing physical and mental tension [21], among other benefits. Currently, some Tuina manipulations have been applied as interventions for children with ASD [22, 23] with a certain degree of success [24]. Qigong Tuina, which is a specific Tuina intervention in children with ASD [25, 26], has been shown to improve the symptoms of children with ASD.

Pediatric Tuina is a traditional Chinese medicine (TCM) therapy that acts on specific acupoints on the hands, back, and arms [27], including Wujing acupoints for different fingers. These acupoints target the spleen, liver, heart, lung, and kidney based on the meridian and collateral theory. The order is located on the thread surface of the five fingers in children (the spleen acupoint is located on the thumb). Stimulating different acupoints by specific manipulations has various curative effects.

However, there is no strong evidence regarding the single-use of pediatric Tuina intervention for ASD treatment [28]. The two objectives of this study are (1) to further evaluate the effectiveness of pediatric Tuina as a complementary therapy for children with ASD aged 2–6 years, by comparing it with conventional treatment through a standardized clinical study design, and (2) to promote and facilitate the use of pediatric Tuina in ASD by allowing more children with ASD to benefit from Tuina as a healthy and environmentally friendly physical therapy.

## Methods/design

### Study design

**Fig. 1:**
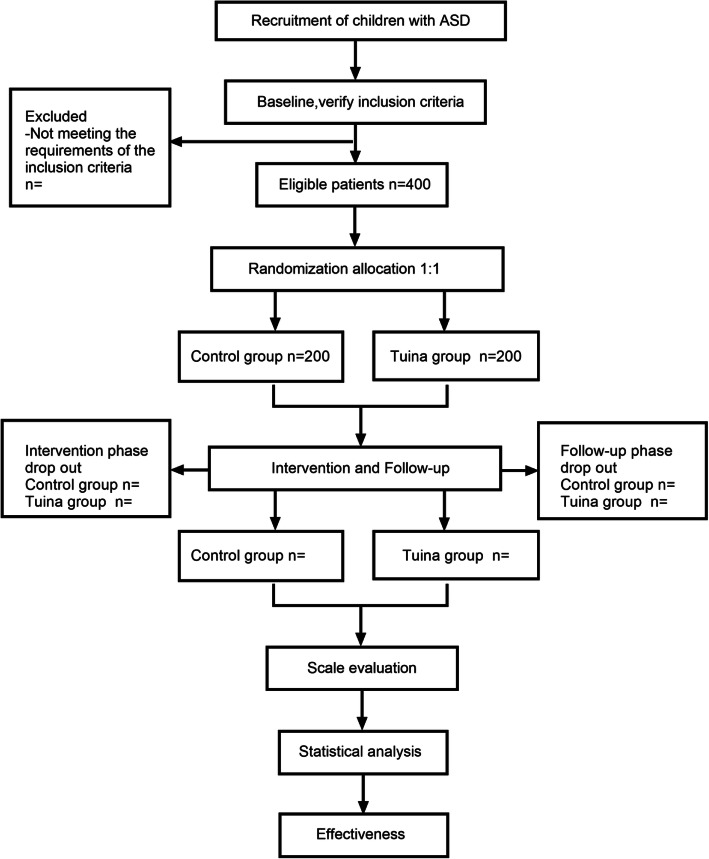
Flowchart of the study. A total of 400 participants will be randomized to the two groups. The interventions will last for 25 min and will be conducted two times per week for 6 weeks, and a 6-month follow-up was performed after treatment. The main efficacy score will be based on the CARS, and the secondary outcome indicators will be evaluated using the ATEC. CARS, Childhood Autism Rating Scale; ATEC, Autism Treatment Evaluation Checklist

This will be a randomized controlled trial. To meet ethical requirements and considering the particularity of ASD, this study will not employ a blind design. The trial will be conducted at Hunan University of Chinese Medicine, China, from November 2020 to December 2022. Eligible children will be randomly divided into the pediatric Tuina plus conventional treatment group or the conventional treatment group based on a computer-generated random table at a ratio of 1:1. Efficacy will be assessed using the Childhood Autism Rating Scale (CARS) and the Autism Treatment Evaluation Checklist (ATEC). Before study commencement, all participants will be assessed using a scale by a third party not involved in the study. The baseline level of the participants will be determined at the time of enrollment; further, the participants will be evaluated again after the 30th treatment, with a follow-up period of 6 months postoperatively. Figure 1 shows the flowchart of the study.

### Participants

This study will recruit 400 children with ASD from autism organizations certified by the Hunan Disabled Persons' Federation, including Xingxueyuan Education Development Center (Tianxin District, Changsha City), Aimeng Rehabilitation Center for Special Children (Yuhua District), and Vitality Autism Rehabilitation Center (Yuelu District), among others.

### Eligibility criteria

#### Inclusion criteria

1. Diagnosis made by a psychiatrist based on the diagnostic criteria for ASD of the Diagnostic and Statistical Manual of Mental Disorders (DSM-5)

2. Age: 2–6 years old

3. CARS score ≥ 30 points

4. The guardian of the child is informed of the study and the child is compliant with the treatment cycles

5. No history of participation in other observational studies or unplanned interventions within the past 30 days, such as acupuncture and herbal treatment that may affect the study results

6. No history of other serious diseases that may affect the outcomes, including organic pathologies, coagulation disorders, congenital heart disease, and epilepsy

7. No history of chronic or acute infections, such as pneumonia

8. No history of other psychiatric disorders, such as ADHD

#### Exclusion criteria

1.Children who are resistant to the Tuina intervention, with persistent crying and inability to adapt

Children who during the study develop conditions like phlebitis, open wounds, and skin breakdown in the manipulated area

#### Elimination criteria

1. Children who do not complete 30 intervention sessions following the research protocol

2. Children who received other interventions that may affect the study results

#### Drop-out criteria

1. Children experiencing adverse events during the trial, such as pneumonia and skin disease, and who could not adhere to the Tuina therapy.

2. Children whose guardian perceived the treatment as ineffective and therefore terminated the treatment.

3. Children withdrawing from the treatment at the original rehabilitation institution or after being transferred to a different rehabilitation institution.

### Interventions

#### Control group

Based on the training of the autism organization mentioned above in this protocol, the control group will strictly follow the five schedule sessions of applied behavior analysis training, five key skill training sessions, five music group training sessions, five oral muscle training sessions, and five sensory training sessions per week. Only teachers with a background in special education will participate in the study.

#### Tuina group

The Tuina group will receive a total of 30 sessions of pediatric Tuina (five sessions per week for 6 weeks, and a follow-up session at the 12th week after the end of treatment). From the perspective of humanistic care, to make the child feel safe, the child will be held by the parents or relatives during the session and placed in a sitting position. Tuina will be performed face to face. When performing acupoint Tuina on the back, the child will lie on his parents or relatives in a prone position.

To standardize the Tuina manipulation, only certified Chinese medicine physicians with 3 months of training in Tuina techniques will perform the procedures. The acupoint prescriptions are mainly based on the TCM theory of the relationship between *Du Meridian* and the *Brain*. After gathering clinical evidence and soliciting the opinions of experienced Tuina experts, we have summarized the following acupoints and manipulation times of Tuina. Table 1 presents the detailed information.

**Table 1:** Tuina acupoints, manipulation times, and methods

Acuponits	Location	Methods	Times
Tianmen	Located from the middle of the eyebrows to the front hairline in a straight line	Linear-pushing with the finger	24
Kangong	Located on a horizontal line connecting both brows	Wiping with the finger	24
Taiyang (temple)	Flat part at each side of the forehead	Pressing-kneading with the finger	24
Wangu (GB12)	Located in the posterior inferior depression of the mastoid behind the ear and above the attachment of the sternocleidomastoid muscle		24
Yamen (GV15)	Located on the nape, 0.5 Cun above the middle of the posterior hairline, and below the first cervical vertebra.		100
Fengfu (GV16)	Located on the nape, when the posterior hairline is straight up 1 Cun, the extraoccipital protuberance is straight down, and the depression between the trapezius muscles		100
Baihui (GV20)	Located on the head, 5 Cun above the middle of the front hairline.		100
Shuigou (GV26)	Located on the face, at the intersection of the upper 1/3 and middle 1/3 of the sulcus		100
Du Meridian	Located on the midline of the back. Tuina therapy is mainly performed from the thoracic spine to the sacral spine.	Pressing-kneading with the finger	20
		Pinching with the finger	20
		Pushing with the finger	300
Jianjing (GB21)	Located under the spinous process of the seventh cervical vertebra, on the midpoint of the line with the acromion	Grasping with the finger	10

### Outcome assessment

The results will be evaluated by a third party otherwise uninvolved in the study. This study will be conducted at the Mental Health Center of the Second Xiangya Hospital of Central South University, Hunan Province, China.

#### Primary outcomes

The main efficacy score will be based on the CARS, which covers interpersonal relationships, imitation, and other 15 aspects, with a total score of 60 points. Each aspect will be scored using a four-point scale: age-appropriate (score 0), mildly abnormal (score 1), moderately abnormal (score 2), and severely abnormal (score 3). Participants with a score < 30, 30–35, and ≥ 36 are considered as non-autistic, moderately autistic, and severely autistic, respectively. Outcomes will be evaluated after the 30th treatment session. Follow-up efficacy assessment will be performed at 6 months post-treatment. The difference in CARS before and after the intervention will be considered significant, effective, and ineffective if the reduction is greater than 10, 5–10 (inclusive), or less than 5 points, respectively [29]..

#### Secondary outcomes

The secondary outcome indicators will be evaluated using the ATEC, which includes four aspects: (1) speech/language/communication, (2) sociability, (3) sensory/cognitive awareness, and (4) health/physical/behavior [30]. With a total of 77 items, each item is rated using a 3-point scale (never, sometimes, and always; recorded as 0, 1, and 2 points, respectively) or a 4-point scale (none, mild, moderate, and severe; recorded as 0, 1, 2, and 3 points respectively). The main purpose of this study is to clarify the improvement in children with ASD after Tuina therapy from different perspectives. The evaluation time points for this outcome are consistent with those of the primary outcomes.

#### Safety assessment

Although pediatric Tuina is a nature-based approach to healing without side effects, some adverse events (AEs) may occur during the treatment process due to medical errors. Skin damage is the most common AE during Tuina. To prevent AE occurrence, strict monitoring will be conducted. In the case of AEs, the researchers will record the time and severity of the AE and promptly deal with it.

### Participant timeline

**Fig. 2:**
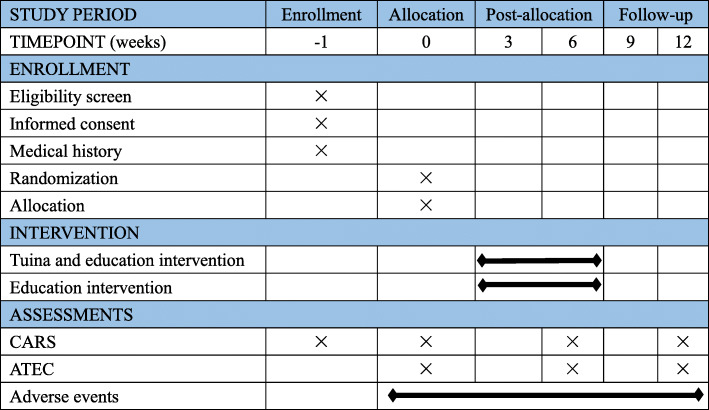
Study schedule for recruitment, interventions, outcome measurements

We have designed the schedule for the recruitment, intervention, evaluation, and follow-up of the participants, as shown in Fig. 2.

### Sample size estimation

The sample size calculation for this study should use a superiority test with two independent sample means to test the scientific hypothesis that both Tuina and conventional treatment are effective for treating children with ASD, but the efficacy of the Tuina treatment may be superior. According to the primary outcome, a difference of more than 5 in the CARS from before to after the intervention is considered to indicate effectiveness [29]. In our initial clinical study [31], the mean value of the difference in CARS from before to after the intervention was 7.5 in the Tuina group (*n* = 22) and 4.27 in the control group (*n* = 22), with a combined standard deviation of 4.43. The test of superiority was a one-sided test, with the test level set at *α* = 0.025 and *β* = 0.2, power=0.8 The ratio of sample size between the two groups was set at a ratio of 1:1, and the shedding rate was 10%. The above data were entered into the interface of “Sample Size Calculation” of the Meisi Medical APP (Shanghai Meisi Medical Technology Co.). Based on the results, 108 cases in each group were deemed necessary. This research is funded by the Hunan Provincial Department of Science and Technology and other institutions that seek to benefit children with ASD. Therefore, we plan to include 200 cases in each group, for a total of 400 cases.

### Recruitment

To secure an adequate sample, subjects in this study will be recruited mainly from rehabilitation institutions for special children in Changsha. The sentinel rehabilitation institutions have a certain stability and are not easily dislodged. Therefore, we will mainly start from the contact institutions. We will obtain information of the relevant institutions by referring to the official website of the Hunan Disabled Persons’ Federation (http://www.hndpf.org/news/tzgg/202012/t20201228_14086695.html). We will then explain the study details to the person in charge of each institution through telephone communication and, after obtaining consent, visit the institutions and sign a cooperation agreement. The person in charge of the institution will set up contact between us and the parents of the children and publish the recruitment notice through WeChat groups and recruitment posters, and eligible children will be enrolled through the application. After the third-party institution determines which children are eligible for inclusion, we will present the study design at the institution to help the guardians of the affected children understand the purpose and significance of the study. Following this, the guardians will be asked to sign an informed consent form before inclusion of their child in the study.

### Allocation

A computer-generated random number table will be used by statisticians otherwise not involved in the study. After completing the basic data assessment of all children with ASD, the children will be randomly assigned to the Tuina and control groups in a 1:1 ratio. The treatment sequences will be provided to each Tuina physician in an opaque envelope, and only the Tuina physician will be aware of the grouping. The third-party assessors as well as the data analysts will not be aware of the treatment allocations.

### Data collection and management

All parents of children with ASD participating in the project will sign the informed consent form and complete the case report form. Finally, a data file will be established and will be kept and collected by a dedicated person from the Data Committee of the First Affiliated Hospital of the Hunan University of Chinese Medicine. It is important to emphasize that data custodians must maintain their independence and access and audit the data on a regular or irregular basis, thus ensuring that no one can tamper with the data. Inspectors will regularly check the informed consent form, research protocol, and evaluation scale to ensure the quality of the clinical research. Regarding the authenticity of the study, all involved researchers will be blinded to the data. Based on the data disclosure requirements of the *CHICTR*, the data of this study will be uploaded to the Baidu Cloud Disk or the website of the School of Acupuncture-Moxibustion and Tuina of Hunan University of Chinese Medicine (zjtn.hnucm.edu.cn) within 6 months after the study completion and will be made publicly available. The premise is that the main research results will be published. All nonmembers should sign an agreement form before accessing the research results.

### Statistical analyses

All data will be analyzed using SPSS (version 21.0; IBM Corp., Armonk, NY, USA); further, all analyses will be conducted using a two-sided test. Normally and non-normally distributed measurement data will be analyzed using the *t*-test and rank-sum test, respectively. The significance level will be set at 5% [32].

### Ethics

To protect the legitimate rights and interests of all our project participants, we will apply for a clinical research ethics review. This study has been approved by the Ethics Committee of the First Affiliated Hospital of Hunan University of Chinese Medicine (HN-LL-KY-2020-020-01).

### Protocol amendments

Any significant protocol revisions will be submitted to the Ethics Committee of the First Affiliated Hospital of Hunan University of Chinese Medicine for approval. After obtaining approval, the changes will be updated on *CHICTR*.

### Ancillary and post-trial care

Tuina is a safe and effective intervention with low probability of AEs, but accidents might occur during the study. If a child with ASD encounters an adverse event during the trial, we will report it to the ethics committee of the First Affiliated Hospital of Hunan University of Chinese Medicine, and a committee of medical experts will determine whether the adverse event is related to the trial. We will also provide treatment costs and financial compensation for study-related injuries based on the actual situation.

#### Dissemination policy

The results of this study will be disseminated in multiple formats after statistical analysis. They will be published in advanced peer-reviewed journals related to ASD, TCM, or complementary alternative medicine research, or in the form of scholarly reports.

## Discussion

ASD development is irreversible, with some symptoms persisting throughout life. Given that improving the quality of life of children with ASD is the common pursuit of all treatments, we should actively explore traditional and non-traditional alternatives to identify the most suitable treatment for ASD.

TCM contains profound wisdom from Chinese philosophy and medical knowledge. Consistent with modern medicine, TCM considers the brain to control emotions, consciousness, and thinking. However, in this theory, the brain is not just the organ in charge of those capabilities, as it has several other functions. The cause of autism is related to the brain; this is a consensus in academic circles. Specific acupoints can improve brain function and promote nerve development. *Du Meridian* is a special meridian closely associated with the physical location and function of the brain [33]. A large part of the *Du Meridian* is located on the midline of the back and front of the head; further, it is directly connected to the brain and gathers the Yang qi of the whole body. *Du Meridian* is an important channel for the output of Jing qi [34], as it serves as a bridge between the brain and other meridians for communicating qi and blood [35]. Therefore, the normal operation of the function of the *Du Meridian* can improve the role of the brain; further, children with ASD have problems with obstruction. In such cases, external stimulation can be performed through Tuina. The formulation of this study protocol will be conducted under the guidance of the *Du Meridian*.

The acupoints and treatment plan for Tuina will be based on TCM. There are several fixed routines in Chinese pediatric Tuina, including starting from the head, which is known as Kaiqiao, for unblocking the body. Four fixed acupuncture points will be used in this study: Tianmen, Kangong, Taiyang (Temple), and Wangu (GB12). The number of operations for each acupuncture point is 24, which corresponds to the Chinese 24 solar terms. The *Du Meridian* is also distributed on the head, with the four representative acupoints including Yamen (GV15), Fengfu (GV16), Baihui (GV20), and Shuigou (GV26). Therefore, they were chosen as the main Tuina points. The back, from the thoracic spine to the sacral spine, is the main circulation part of the *Du Meridian* and the main stimulation point. Three methods are used for stimulation: pressing-kneading manipulation, pinching, and pushing with the finger. The last step is grasping Jianjing (GB21), also known as Guanqiao, which relaxes the body.

However, the efficacy of Tuina in ASD treatment requires further investigation. This clinical randomized controlled trial will compare the effects of Tuina combined with behavioral and single education interventions on the rehabilitation of children with ASD. The CARS will be used to evaluate its efficacy, although our goal is to improve the core ASD symptoms in children, including the improvement of intestinal symptoms. This research plan could help build a strong basis for the application of Tuina therapy for children with ASD, which could allow more patients to benefit from this treatment.

## Study limitations

First, the diagnosis of ASD will not be made using the Autism Diagnostic Interview-Revised or Autism Diagnostic Observation Scale due to copyright restrictions; instead, the DSM-5 will be used.

Regarding the evaluation, there are no objective biomarkers for evaluating the efficacy of ASD treatments. This study will employ a scale to evaluate the efficacy of the ASD treatment, which may lead to subjective errors.

Additionally, it is impossible to blindly perform Tuina therapy. Furthermore, the standardization of Tuina techniques and stimulation amount varies from person to person. There will inevitably be some differences in specific operations. To reduce such errors, only Tuina therapists with rigorous training and professional qualifications will participate in the study to ensure standard treatment.

Finally, given the ethical requirements, it is not possible to prohibit all children from participating in other educational or behavioral interventions. Therefore, a single Tuina intervention trial is insufficient to evaluate its feasibility for clinical application.

## Trial status

The recruitment of patients for this clinical trial, which started in November 2020 and ended in September 2021, it is now entering the clinical intervention phase. The trial was registered in the Chinese Clinical Trial Registry on 28 November 2020 (registration number: ChiCTR2000040452).

## Data Availability

The results of this trial will be presented in peer-reviewed journals.

## Abbreviations

AE: Adverse event
ADHD: Attention deficit hyperactivity disorder
ASD: Autism spectrum disorder
ATEC: Autism Treatment Evaluation Checklist
CARS: Childhood Autism Rating Scale
DSM-5: Diagnostic and statistical manual of mental disorders
TCM: Traditional Chinese medicine
